# Air Health Trend Indicator: Association between Short-Term Exposure to Ground Ozone and Circulatory Hospitalizations in Canada for 17 Years, 1996–2012

**DOI:** 10.3390/ijerph15081566

**Published:** 2018-07-24

**Authors:** Hwashin Hyun Shin, Wesley S. Burr, Dave Stieb, Lani Haque, Harun Kalayci, Branka Jovic, Marc Smith-Doiron

**Affiliations:** 1 Environmental Health Science and Research Bureau, Health Canada, Ottawa, ON K1A 0K9, Canada; Dave.Stieb@Canada.ca (D.S.); Lani.Haque@Canada.ca (L.H.); Harun.Kalayci@Canada.ca (H.K.); branka.jovic@canada.ca (B.J.); marc.smith-doiron@canada.ca (M.S.-D.); 2 Department of Mathematics and Statistics, Queen’s University, Kingston, ON K7L 3N6, Canada; 3 Department of Mathematics, Trent University, Peterborough, ON K9L 0G2, Canada; wesleyburr@trentu.ca; 4 School of Epidemiology and Public Health, University of Ottawa, Ottawa, ON K1N 6N5, Canada

**Keywords:** Bayesian hierarchical model, cerebrovascular disease, hospitalizations, ground-ozone, ischemic heart disease, other heart disease, public health risk, trend

## Abstract

The Air Health Trend Indicator is designed to estimate the public health risk related to short-term exposure to air pollution and to detect trends in the annual health risks. Daily ozone, circulatory hospitalizations and weather data for 24 cities (about 54% of Canadians) for 17 years (1996–2012) were used. This study examined three circulatory causes: ischemic heart disease (IHD, 40% of cases), other heart disease (OHD, 31%) and cerebrovascular disease (CEV, 14%). A Bayesian hierarchical model using a 7-year estimator was employed to find trends in the annual national associations by season, lag of effect, sex and age group (≤65 vs. >65). Warm season 1-day lagged ozone returned higher national risk per 10 ppb: 0.4% (95% credible interval, −0.3–1.1%) for IHD, 0.4% (−0.2–1.0%) for OHD, and 0.2% (−0.8–1.2%) for CEV. Overall mixed trends in annual associations were observed for IHD and CEV, but a decreasing trend for OHD. While little age effect was identified, some sex-specific difference was detected, with males seemingly more vulnerable to ozone for CEV, although this finding needs further investigation. The study findings could reduce a knowledge gap by identifying trends in risk over time as well as sub-populations susceptible to ozone by age and sex.

## 1. Introduction

Considerable research over the last 20 years has established a clear and significant link between ground-level ozone (ozone hereafter) concentrations and adverse health effects, including asthma [1], heart attacks and strokes [2,3], and the most severe outcome, death [4]. Early research, largely consisting of cohort studies linked these health effects to long-term ambient ozone exposure [5,6,7]. Continued efforts led to evidence of links between short-term exposure to ozone and several health markers [8], including myocardial infarction and ischemic stroke [2,9,10,11], cardiovascular and respiratory diseases [12,13], and mortality [4,14,15].

Until the year 2000, studies linking ozone to human health dealt primarily with long-term exposures [16]. This was mainly due to two reasons: lack of suitable data in hourly or daily measurement, and lack of suitable analytical frameworks. It was not until the invention of Generalized Additive Models (GAMs) in 1986 [17], and their subsequent popularization in 1990 [18] (along with the development of suitable software tools for estimating such models) that sufficient structure was in place to allow for the estimation of the risk due to acute exposure. The first substantive work in this vein that truly leveraged the power of GAMs was that of Dominici et al. [19].

Since 2000, there has been a tremendous amount of work published on the relationship between acute exposure to air pollution (AP) and a variety of health outcomes. These outcomes include mortality [20,21], ischemic stroke [2,22,23], cardiac hospitalizations [24,25] and general hospitalizations [26,27]. Many of these studies use time-series regression models, typically computed in the framework of GAMs or Generalized Linear Models (GLMs). A risk assessment of Health Canada [28] concluded that there was a suggestion of a causal relationship with cardiovascular-related health outcomes. A more recent assessment by the US Environmental Protection Agency (EPA) [29] reported cardiovascular health outcomes as likely to be causally related to exposure to ozone in addition to respiratory health outcomes.

Additionally, most of such studies use one contiguous time block for analysis (the static approach), such blocks including 1987−2000 (the NMMAPS database, [18]) or 2000−2005 (the French heat wave of 2003, [30]). An exception to this rule (the dynamic approach) has been attempted by Shin et al. [31,32] in the course of developing the Air Health Indicator (AHI), which seeks to model time trends [21,33] in annual health risks rather than overall risk from all years available combined.

Daily hospitalization varies dynamically over space and time in response to changes in daily air quality and other factors. Hundreds of time series studies worldwide on the association between both daily mortality and hospitalizations (or morbidity), and daily air pollution concentrations have been published from the two perspectives of static and dynamic approaches. The static approach assumes the association is constant over time [14,30]. In other words, the current impact of air pollution on hospitalization would be the same as 10 or 20 years ago. By contrast, the dynamic approach considers the association as influenced by environmental, social, or medical changes over time. As discussed in Shin et al. [21,33], there are several potential reasons for temporal changes in the impact of air pollution on public health:

- The population most at risk for hospitalization related to air pollution may change over time.
- The effect modifiers such as heart or lung related health care including medical treatments and medicines may change over time.
- The association between exposure and hospitalization may be non-linear.

It is thus expected that the dynamic approach will allow us to test the assumption of the static approach and add more information, as compared to the static approach where all the assumptions remain the same. In addition, the dynamic approach is able to show temporal trends over time such as a linearly increasing, decreasing or nonlinear trend. The trend can be used in AP-related regulations assessment or inform accountability studies seeking to evaluate the effectiveness of regulatory measures in influencing specific health endpoints.

Model-dependency of risk estimates has been a critical issue in risk estimation, since different models may return non-negligible differences in risk estimates. The degree of model-dependency, however, could be of less concern for time trend estimation as long as the same models are applied to each time window within the study period. As long as a reasonable model has been selected after an extensive sensitivity analysis, it can be considered a measure for the association between ozone and particular health outcome relevant to public health each year, providing internally consistent trends in the annual seasonal associations.

The Air Health Trend Indicator (AHTI), formerly known as AHI, is part of the Canadian Environmental Sustainability Indicators program. It is designed to estimate public health risk related to short-term exposure to air pollution and to detect trends in the annual national health risks. The objectives of this study are threefold. Firstly, we apply a multi-year estimator [31,32] and a newly proposed time smoother (SLP [34]) for annual associations between ground-level ozone and three specific types of circulatory hospital admissions. Secondly, we estimate the short-term associations by various factors: season, lag of ozone, age and sex. Finally, we examine temporal changes in those annual risks at the national level to provide the AHTI for circulatory hospitalizations over 17 years from 1996 to 2012.

## 2. Materials and Methods

### 2.1. Circulatory Hospitalizations

Circulatory hospitalizations account for approximately 16% of all non-accidental hospitalizations in Canada. This study examined three specific types of circulatory causes, covering 85% of all circulatory hospitalizations, classified by the International Classification of Diseases (ICD) 10 version: ischaemic heart disease (IHD, I20–I25, 40% of circulatory hospitalizations), other heart disease (OHD e.g., pericarditis, endocarditis, myocarditis, cardiomyopathy, valve disease, conduction disturbances, arrhythmias, heart failure, I30–I52, 31%) and cerebrovascular disease (CEV, I60–I69, 14%). The other circulatory causes did not have enough daily hospitalization admissions and thus were excluded from the study. The three specific types of hospitalizations can reasonably have associations estimated for exposure to ozone for urban Canadian communities at the census division (CD) level for 17 years from 1996 to 2012, based on the communities each having a reasonably complete time series of adequate data (i.e., population of the CD is not too small) for daily hospitalization counts of the three specific types of circulatory causes and also air pollution measurements for the time span of interest.

Association between daily variations in ozone and those in the three specific types of hospitalizations, respectively, was estimated by 5 groups based on age and sex, and denoted in their recorded (database) naming scheme: (1) fm1+ (called “base group” of both sexes aged ≥ 1 year); (2) fm50+ (“over50 group” of both sexes aged > 50 years); (3) fm65+ (“seniors” of both sexes aged > 65 years); (4) f1+ (“females” aged ≥ 1 year); and (5) m1+ (“males” aged > 1 year). Since about 90% of the circulatory related hospitalizations over the study time period was aged 50 and over, the over50 group was defined to exclude younger patients. Due to the small difference (<10%) in patients between the base group and over50 group, two groups are expected to show little differences in associations.

### 2.2. Spatial Coverage

**Figure 1:**
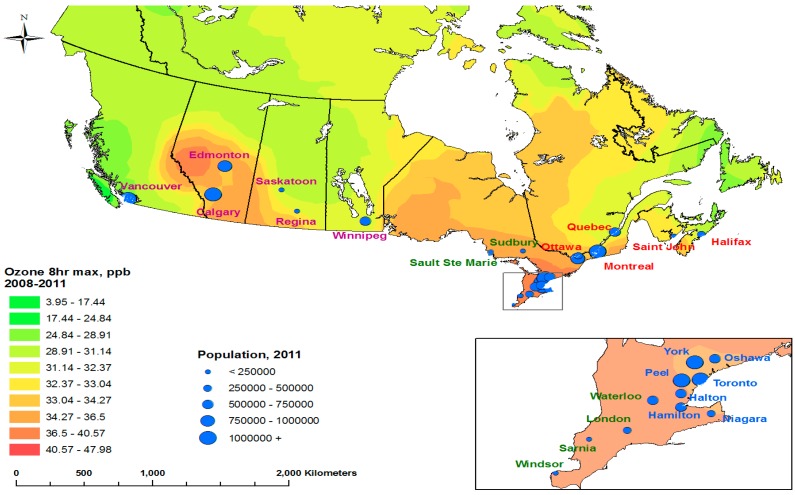
Selected Canadian Census Divisions with population size and spatially interpolated ozone concentrations.

Twenty-four urban CDs were the selected geographic locations displayed in Figure 1. Each community’s geographic boundaries were defined by the CD associated with the communities, determined by Statistics Canada. Next to provinces and territories, CDs are the most stable administrative geographic areas, and are therefore used for this study. Taken together, the 24 urban CDs cover 52−54% of the Canadian population over the study time period. For each community, there were three classes of data used: daily ozone concentrations, daily counts of circulatory hospitalizations, and daily potential confounders for the ozone-hospitalization associations.

### 2.3. Data Sources

#### 2.3.1. Ozone Concentrations

The daily ozone concentration data were obtained from the National Air Pollution Surveillance (NAPS) system (Environment Canada, 2013). Established in 1969, the NAPS network provides accurate and long-term air quality data of a uniform standard across Canada, intended to monitor the quality of ambient (outdoor) air in populated regions, using specific procedures for the selection and positioning of monitoring stations.

For the metric of air pollution (ozone), the daily maximum 8-hour average concentration was selected as it has been used for the Canadian Ambient Air Quality Standards (CAAQS) and US National Ambient Air Quality Standards (NAAQS). For each NAPS monitoring station, the daily maximum 8-hour average concentration for a certain day was considered available only if at least 75% of the 8-hour average and/or hourly concentrations for that day were available; otherwise, the concentration was recorded as missing. For each census division, the daily maximum 8-hour average concentration was averaged over monitoring stations if there were 2 or more stations located in that census division. The annual Canadian national-level ozone concentrations were obtained as population-based weighted averages (*O*_3_*.w*) for all communities included in the analysis as follows:

$$O_{3}.w=\sum_{i=1}^{24}w_{i}\times O_{3i}$$

where wi=(pop. of community i)(total pop.) and $O_{3i}$ is ozone concentration of community i. The annual national weighted averages of ozone concentration by this method reflect the average aggregate (total) population exposure. 

#### 2.3.2. Daily Counts of Cause-Specific Hospitalization

The daily counts of cause-specific non-accidental hospitalizations were obtained from the national morbidity database managed by the Canadian Institute for Health Information (CIHI). Based on the International Classification of Diseases (ICD) the hospitalization data included only hospitalizations from internal causes excluding external causes such as accidents or injuries. During the study time period, the ICD codes have changed from the 9th to the 10th version. To be consistent, we used a conversion table from ICD-9 to ICD-10, which was provided by CIHI, and applied to the data for years prior to the formal health system transition. This process is critical to the AHTI, as it focuses temporal trends in adverse health effects of ozone on population. 

Regarding cause-specific hospitalizations in particular, our primary interest was in heart or blood flow interruption related disease hospitalization. For this specification, our hospitalization data were categorized by the heart disease group (ICD-10 code between I00–I99, [35]). The three circulatory hospitalizations chosen (above) were extracted by the ICD-10 codes: IHD by I20–I25, OHD by I30–I52, and CEV by I60–I69.

The circulatory hospitalization data were extracted for a specified CD only when the CD of residence of the patient was the same as that of the location of hospitalization occurrence, for all years from 1996 through 2012, i.e., those patients who lived in a different CD from their hospitalization CD were excluded. This process is also necessary to reduce misclassification in ozone concentration assignment, as this study is based on the ecological design, where the same concentrations were assigned for all residents within a CD.

#### 2.3.3. Potential Confounders to the Hospitalization-Air Pollution Association

For potential confounding variables in the ozone-hospitalization association, three factors were considered: calendar time, temperature, and indicators for days of the week. Calendar time is included to control both long-term and seasonal variations, and to account for unmeasured slowly varying confounders such as demographic shift. Daily temperature is included to control for the short-term effect of weather on daily hospitalization, and the day of the week accounts for hospitalization that varies by day of the week (DOW). Daily mean temperature in Celsius was obtained from the National Climate Data and Information Archive [36]. As lifestyle factors such as smoking or cholesterol do not vary meaningfully from day to day, they are not included as confounders beyond the use of the smooth function of calendar time which should account for their slow demographic changes.

### 2.4. Annual Hierarchical Model

One of the main purposes of this study is to identify trends in the association between ozone exposure and circulatory hospitalization at national levels based on city-specific annual hospitalization risks. The annual national risks of circulatory hospitalization were obtained using a Bayesian hierarchical model.

First, city-specific risks were modeled using an over-dispersed Poisson GAM (Equation (1)). The annual city-specific risks were estimated by applying a rolling multi-year estimator using the current year and the 6 most recent previous years. Seven years were used as in the simulation study conducted by Shin et al. [31]. As an example, the national risk for the year 2009 was obtained using a total of seven years from 2009–2003 with initial unequal weights generated by the tricube weight function *K(u)*, i.e., 0.220, 0.218, 0.205, 0.172, 0.118, 0.056, and 0.011 applied in decreasing temporal order. Formally, $K\left(u\right)=\alpha {\left(1-{\left(\frac{u-1}{7}\right)}^{3}\right)}^{3},\;u\in \left\{1,\;2,\;\ldots ,\;7\right\},$ with α chosen to cause the sum of the weights to add to 1. The contribution of these seven years was thus weighted such that there was a greater contribution from years closer to the current year. The multi-year estimator was employed to emphasize the direction of a trend in the annual national hospitalization risk estimates by smoothing out noisy fluctuations. For ozone, this approach provides annual risk estimates beginning in 2002, as the first year is 1996.

Second, the annual national circulatory hospitalization risks were estimated by pooling the city-specific risk estimates (Equation (2)). The mathematical model descriptions are as follows: For city i and year j, the true city-specific risks $\beta_{ij}$ were assumed to fluctuate randomly around their respective national risks $\mu_{j}$ with variance $\omega_{j}^{2}$, which models the heterogeneity among the cities (between-city spatial variation) for a given year *j*. While the city-specific risks $\beta_{ij}$ were modeled as normally distributed with mean $\mu_{j}$ and variance $\omega_{j}^{2}$, the city-specific estimates $\hat{\beta_{ij}}$ were modeled as normally distributed with mean $\beta_{ij}$ and sample variance $v_{j}^{2}.$

(1) $$\log\left(E\left[Y_{ij}\left(t\right)\right]\right)=\beta_{ij}\left(t\right)\times x_{ij}\left(t\right)+f_{j}\left(t\right)+g_{j}\left(temp\left(t\right)\right)+DOW_{j}\left(t\right)$$

(2) $$\beta_{ij}\;|\;\mu_{j},\;\omega_{j}^{2}\;∼N\left(\mu_{j},\;\omega_{j}^{2}\right),$$

where, on day *t*, *Y(t)* is the daily count of hospitalizations (response), *x(t)* the ambient air pollutant (predictor of interest), *f(t)* the smooth function of time (for days *t = 1*, *2*, *3*, *…*, *T*), *g(temp(t))* a smooth function of temperature, and *DOW(t)* a factor variable representing the day of week.

We implemented a Gibbs sampler (through WinBUGs), which is a Markov Chain Monte Carlo (MCMC) method for obtaining large numbers of random samples from the posterior distributions of the national risks, because direct sampling is practically impossible. A full description of this hierarchical model protocol has been previously provided in more detail in [33]. The statistical computing language and environment R 3.4.1 [37] was used for all computations.

### 2.5. Sensitivity Analysis

Consensus on a good national-level model for ozone-hospitalization association can be difficult to achieve due to considerable sensitivity of risk estimates to both the model and the priors used in the hierarchical aggregation. Among the confounders discussed above, ambient temperature has a potentially significant effect on the impact of ozone on circulatory hospitalization since temperature and ozone are correlated and share short-term patterns quite strongly. The smallest time unit in this study is one day. Combining all 17 available years from 1996 to 2012, we thus considered the impacts of temperature at lags 0, 1 and 2 and ozone at lags 0, 1 and 2. To illustrate the effects of temperature, we also considered another model without the temperature term. As a result, we investigated a total of 12 models representing the combinations from 4 different temperature options and 3 different ozone options.

In addition, seasonal differences in ozone’s impact on circulatory hospitalizations were also examined by three time-windows: a warm season between April and September, a cold season between October and March, and year-round for the period January to December. 

As for the prior for the variance $w_{j}^{2}$, i.e., the between-city spatial variation in Equation (2), we considered a uniform distribution (denoted by U(0,α]) as a non-informative prior after exploring a number of alternatives. To examine the influence by α, we explored national risk estimates with α=0.1, 1, 100.

## 3. Results

During the study time period we found higher associations for warm season between 1-day lagged ozone and the three specific types of circulatory causes, respectively, which were not sensitive to the prior uniform distribution, comparing to negligible associations from other season (cold season and year-round) and other lag of ozone (no lag and 2-day lag). We thus report findings for warm season 1-day lagged ozone. We first examined trends in annual population, circulatory hospitalization, IHD, OHD, CEV, and population weighted ozone concentration. The associations between ozone and the three specific types of circulatory hospitalizations, respectively, were estimated for all years combined (static analysis) to give a base group overall association, and by the 7-year rolling estimator (dynamic analysis) to find any existing trends. 

### 3.1. Trends in Annual Data

#### 3.1.1. Population and Hospitalization

**Figure 2:**
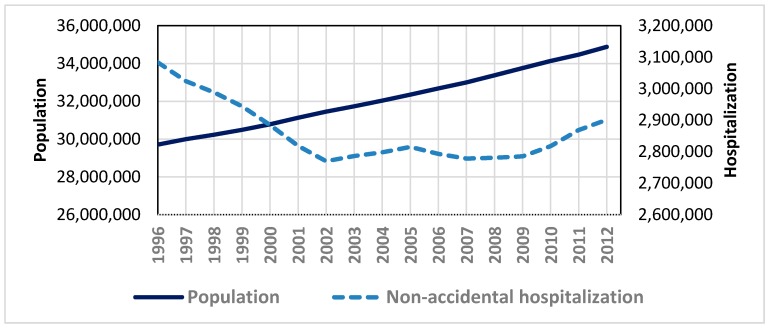
Trends in total population and non-accidental hospitalization counts (in persons) between 1996 and 2012, Canada: solid line for population using left y-axis; dashed line for hospitalizations using right y-axis.

During the study time period for 17 years between 1996 and 2012, total aggregate non-accidental hospitalization counts decreased by 7% from 3.1 to 2.9 in millions, while Canada’s population increased by 17% from 30 to 35 in millions, with a 5% increase in the subpopulation of seniors (aged 65 and over). Despite the increased numbers of seniors, the hospitalization rate (by total population) decreased by 2% from 10% to 8%. This implies that the Canadian population was aging, but simultaneously was becoming healthier, at least in terms of hospitalizations. For example, we have observed about 25% and 20% decline in the total aggregate IHD and CEV hospitalizations, respectively, over the study period. While the population displayed a gradually and monotonically increasing trend, hospitalizations demonstrated a mixed trend with a steady decrease until 2002, roughly flat until 2010, and then slight increases for recent years (Figure 2).

For the 24 urban CDs in Figure 1, Table 1 summarizes overall changes in demographic distribution and Table 2 the same for circulatory hospitalizations (IHD, OHD, and CEV). Out of 24 CDs, 21 CDs (88%) show decreased circulatory hospital admissions in general. More specifically, declines were observed in 22 CDs (92%) for IHD, 7 CDs (29%) for OHD, and 19 CDs (79%) for CEV. Therefore, the decline in circulatory hospitalization was led by IHD and CEV, but not OHD.

#### 3.1.2. Trend in Ozone Concentrations

A total of 114 NAPS stations were used in this study. Table 3 summarizes basic statistics on NAPS stations, ozone data availability, range of ozone, and range of temperature for the 24 urban CDs. Ozone data availability is excellent mostly higher than 95% over the study time span. The reason for low availability for Halifax is that there were no ozone measurements for two years, 1997 and 1999, due to monitoring station malfunctions.

**Figure 3:**
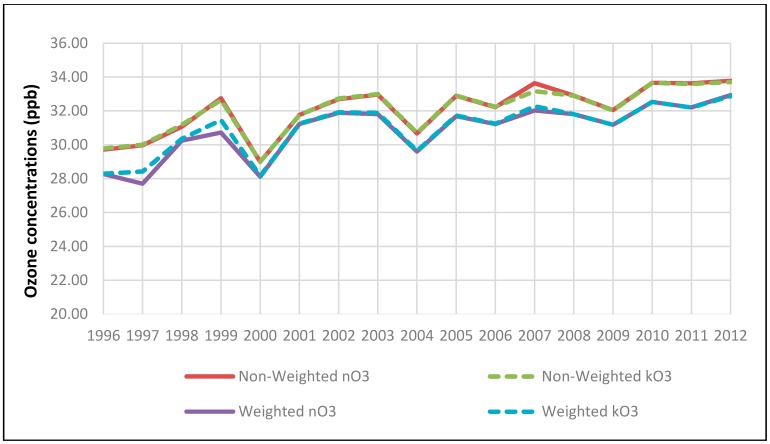
Trends in ozone concentrations in ppb: (**a**) solid lines for non-imputed ozone (nO3), non-weighted (red) versus weighted (purple); (**b**) dashed lines for imputed ozone (kO3), non-weighted (green) versus weighted (blue).

Despite ozone’s good data availability, there were still missing daily data. Considering ozone’s diurnal, weekly, monthly and seasonal variations, we imputed those missing data [38], denoted by kO3 for imputed ozone against nO3 for original unimputed ozone. The imputation method was to implement the gap-filling through spectral estimation and filtering. Combining population-based weights and imputations, we could compare four metrics of ozone concentrations in parts per billion (ppb) as shown in Figure 3. While negligible differences were observed between kO3 and nO3, differences of 1 or 2 ppb due to population weights were clearly noticed. This implies that among the 24 urban cities more populated cities had lower ozone concentrations than less populated cities, which could be related to greater traffic volume and accompanying O3 scavenging accounting for lower O3 concentrations in more populated cities. It was also noticed that ozone concentrations increased by 4–5 ppb overall, indicating 13% increase for unweighted ozone and 18% increase for weighted ozone showing a faster change. In this study the weighted ozone concentration is not used in the analysis; it is used only to examine the trend in exposure nationally, and the imputed ozone (kO3) was used.

### 3.2. Trends in Relative Risk of Circulatory Hospitalizations by Age and Sex

#### 3.2.1. Trend in Ozone-IHD Associations

**Figure 4:**
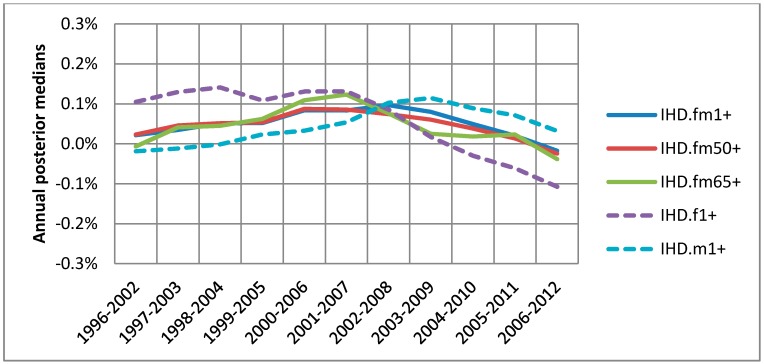
Annual associations between warm season 1-day lagged ozone and IHD hospitalizations for 5 groups: base group (fm1+) in solid blue; over50 group, (fm50+) in solid red; seniors (fm65+) in solid green; females aged ≥1 (f1+) in dashed purple; and males aged ≥1 (m1+) in dashed blue.

For the warm season from April to September, the association between ozone and IHD hospitalization was compared by 5 different groups based on age and sex as described in Section 2. Figure 4 shows the medians of annual posterior distributions for each group, indicating that the probability of the association equal to or larger than the median is 0.5 for each year. Higher positive median value means higher risk of IHD hospitalization in relation with exposure to ozone. For any group tracking those medians provide a tool for trend detection and group comparison. 

Overall there was little difference by age among the 3 groups of all ages (≥1), over50 and seniors but some difference by sex was observed (Figure 4). While the senior subpopulation (fm65+) did not show higher associations than the base group (fm1+), males (m1+) or females (f1+), they showed higher associations for different time periods (Figure 4). For all 5 groups a visible decline in association started from 2009, which appeared to be a turning point in trends over the study time period. It should be noted that males, compared to females, changed from lower to higher association in 2009 onwards as displayed in Figure 4, which show smoothed trends.

**Figure 5:**
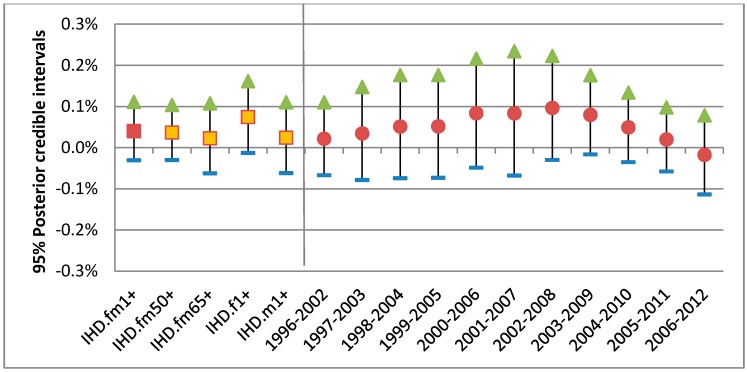
95% posterior intervals for the associations per 10 ppb of 1-day lagged ozone with IHD hospitalizations for warm season: (**a**) 17 years combined risks for 5 groups (squares); (**b**) 11 annual risks of base group (circles in red).

Figure 5 shows the median (middle point) and quantile interval at 2.5% and 97.5% (i.e., 95% posterior interval) of the posterior distribution: all years combined for each group (first 5 intervals) and annual for the base group only (11 intervals on right). While the first 5 posterior intervals show overall ozone-IHD associations as graphical summaries, the annual posterior intervals provide between-city heterogeneity as well as trend. Since national estimates were based on pooling over the 24 urban CDs, the posterior intervals would become relatively wider when their between-city heterogeneity was relatively larger. Increased heterogeneity was observed as the median values became larger.

For the warm season the all-year combined static national risk of the IHD per 10 ppb of 1-day lagged ozone was 0.4% with 95% posterior interval (−0.3–1.1%) for all 17 years combined. Compared with this base group as a reference, only females were at higher risk, with an estimated level of 0.8% with 95% posterior interval (−0.1–1.6%) as shown in Figure 5. The annual 95% posterior intervals of the base group risk of the IHD show a mixed trend of a gradually increasing and then a linearly decreasing change. (The annual 95% posterior intervals of the other 4 groups can be found in Supplementary Figures S1–S4.)

#### 3.2.2. Trend in Ozone-OHD Associations

**Figure 6:**
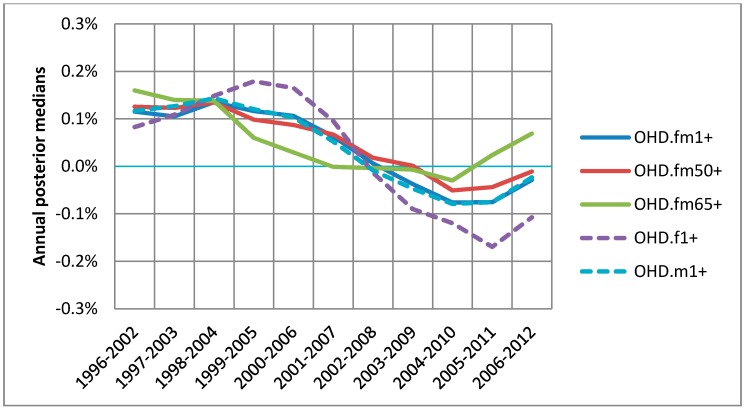
Annual associations between warm season 1-day lagged ozone and OHD hospitalizations for 5 groups: base group (fm1+) in solid blue; over50 group, (fm50+) in solid red; seniors (fm65+) in solid green; females aged ≥1 (f1+) in dashed purple; and males aged ≥1 (m1+) in dashed blue.

For the warm season from April to September, the association between ozone and OHD hospitalization was compared by 5 different groups based on age and sex, as described in Section 2. Overall, some differences by age and sex were observed. While two groups, over50 (fm50+) and males (m1+), did not show higher associations than the base group, the other two groups, seniors (fm65+) and females (fm1+), showed higher associations for different time periods (Figure 6). For all 5 groups a visible decline in association was observed. It should be noted that the females changed from higher to lower association in 2009 onwards, whereas the seniors changed from lower to higher association, which was not observed for the IHD hospitalization.

**Figure 7:**
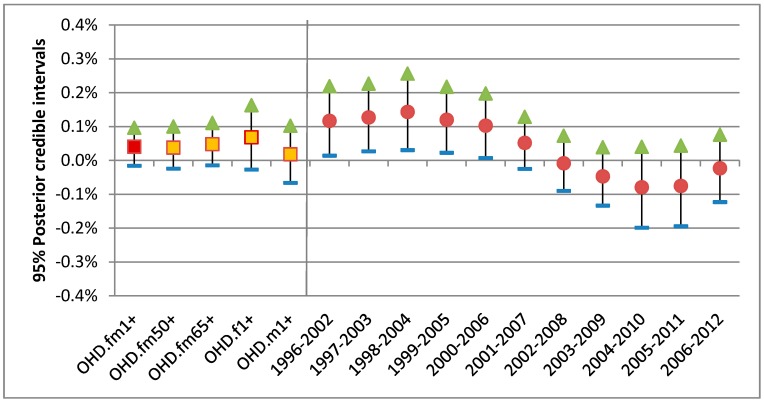
A total of 95% posterior intervals for the associations per 10 ppb 1-day lagged ozone with OHD hospitalizations for warm season: (**a**) 17 years combined risks for 5 groups (squares); (**b**) 11 annual risks of base group (circles in red).

For the warm season the all-year combined static national risk of the OHD per 10 ppb of 1-day lagged ozone was 0.4% with 95% posterior interval (−0.2–1.0%) for all 17 years combined. Compared with this base group, only females were at slightly higher risk of 0.7% with 95% posterior interval (−0.3–1.6%) as shown in Figure 7. The annual 95% posterior intervals of the base group risk of the OHD shows a mixed trend of a flat and then decreasing pattern. (The annual 95% posterior intervals of the other 4 groups can be found in Supplementary Figures S5–S8.)

#### 3.2.3. Trend in Ozone-CEV Associations

**Figure 8:**
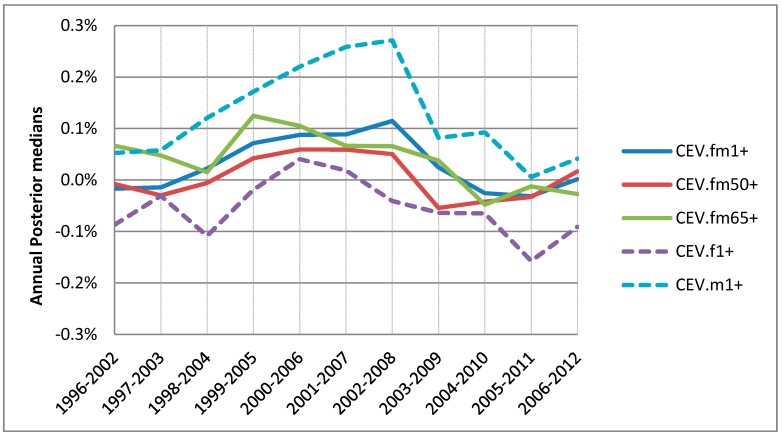
Annual associations between warm season 1-day lagged ozone and CEV hospitalizations for 5 groups: base group (fm1+) in solid blue; over50 group, (fm50+) in solid red; seniors (fm65+) in solid green; females aged ≥1 (f1+) in dashed purple; and males aged ≥1 (m1+) in dashed blue.

For the warm season from April to September, the association between ozone and CEV hospitalization was compared by 5 different groups based on age and sex as described in Section 2. Overall little difference by age but some visible differences by sex were observed. While the senior subpopulation (fm65+) did not show higher associations than the base group (fm1+), males (m1+) and females (f1+) showed higher and lower associations, respectively, consistently over the study time period (Figure 8). For all groups except for males not much annual variations appeared. It should be noted that males, compared to females, were always at higher CEV hospitalization risk with a sudden decline for 2009.

**Figure 9:**
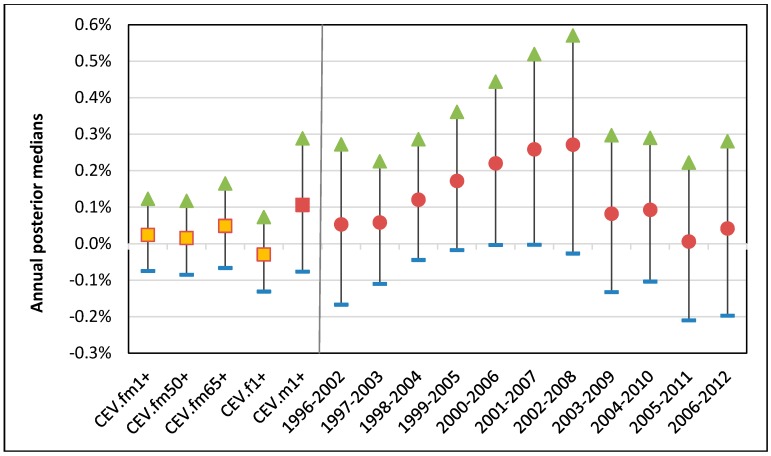
A total of 95% posterior intervals for the associations per 10 ppb 1-day lagged ozone with CEV hospitalizations for warm season: (**a**) 17 years combined risks for 5 groups (squares); (**b**) 11 annual risks of males (circles in red).

For the warm season the all-year combined static national risk of the CEV per 10 ppb of 1-day lagged ozone was 0.2% with 95% posterior interval (−0.8–1.2%) for all 17 years combined. Compared with the base group, males were at higher risk of 1.1% with 95% posterior interval (−0.8–2.9%), which is much wider than that for IHD and OHD, as shown in Figure 9. The annual 95% posterior intervals of the base group risk of the IHD shows a mixed trend of a gradually increasing and linearly decreasing changes. (The annual 95% posterior intervals of the other 4 groups can be found in Supplementary Figures S9–S12).

## 4. Discussion

In this section we will discuss trends in the air health indicator, differences reported in the ozone-hospitalization associations by age and sex, and limitations of the study. 

### 4.1. Air Health Indicator with Trend

A substantial body of research has established that the health risk associated with common air pollutants rises and falls with levels of pollution within a time frame of one to several days. This has been demonstrated for several health markers, particularly heart- and lung-related mortality and hospitalization. For this reason, the Air Health Trend Indicator (AHTI) has been developed as tool to track the impacts of short-term exposure to outdoor air pollution on public health over time.

The proposed model, a dynamic multi-year estimator [31,32,33] (here 7 years), is capable of not only tracking the changes in risk associated with varying pollutant concentrations, but also identifying specific pivoting years for those changes. The changes in risk may result from changes in demographic distribution, population susceptibility (due to better health protection), exposure (due to changes in the built environment), unidentified co-pollutants that fall out of synchronization with the measured pollutant, or any number of additional factors. 

Risks were determined for each calendar year and are based on information over the warm season from April to September when Canadians spend more time outdoors (as compared to the cold season, when low temperatures discourage this) and are thus more exposed to ozone from outdoor sources [21,39,40,41]. Based on this tracking tool, we examined the trend in national-level annual circulatory hospitalizations (in particular, IHD, OHD and CEV) known to be associated with short-term exposure to ozone [2,3,9,25,42]. For the warm season over the study time period of 1996–2012 there were overall gradual declines for OHD, and mixed trends of incline and decline for IHD and CEV in the annual risks for the base group of all ages, both sexes (fm1+). However, the observed trends are not fully explained by external factors such as demographic changes and thus further investigations are needed.

This finding supports the dynamic approach over the static approach, as the year by year changes in the national risks seem unlikely to be completely random fluctuations. This is evidence of potentially large differences in the ozone-linked circulatory hospitalization risk estimates if different study periods are considered. Despite gradually increasing ozone concentrations, the ozone-linked circulatory hospitalization risk estimates displayed a decline for recent years. This leads naturally to interesting questions:

- Is the observed decline true, and not model-related?
- Why do the concentrations and adverse effects of ozone for recent years show opposite time trends?
- Are there some other confounders that led to declines for recent years?
- Further investigations are necessary to be able to answer these questions.

### 4.2. Little Difference by Age

The Canadian population is growing and aging but non-accidental hospitalization rates for the population have decreased from 10% to 8% between 1996 and 2012. The senior subpopulation (ages over 65) comprises 15% of the population but 39% of hospitalizations nationally in 2012: clearly seniors are hospitalized more often than the younger population. This study analyzed the 24 urban cities in Figure 1, which have about 54% of the Canadian population, comprising 12% seniors and 5% non-accidental hospitalization rate (all ages). Therefore, this study is based on a younger (12% vs. 15% seniors) and healthier (5% vs. 8% hospitalization rate) subpopulation, which may lead to unmeasurable bias [43] in estimates for the association between ozone and the three specific types of hospitalizations.

Given the fact that seniors are hospitalized more often than younger generations, we may expect an increased number of hospitalizations as the Canadian population is clearly aging. However, we have observed about 25% and 20% decline in the total aggregate IHD and CEV hospitalizations, respectively, in this study. This may indicate strong effect modifiers such as decreased smoking rates, leading to the significant decline in the IHD and CEV hospitalizations. A few studies have reported inconsistent age difference in the associations between ozone and cardiovascular-related hospitalizations [44,45]. When comparing age groups, while there is evidence of increased associations between ozone and mortality for the senior population compared to the young population, there is still lack of consistent associations between ozone and hospitalizations [46]. The study finding of slight differences by age (below versus above 65 years) suggests that the adverse health effect of ozone on circulatory hospitalization is less likely to depend on age than what has been expected [2,47].

### 4.3. Noticeable Sex-Specific Differences

The data used in this study has information on biological attributes only and thus our analyses analyzed sex, not gender. Females are about 51% of the Canadian population and 57% of the hospitalizations, which indicates females were admitted to hospitals slightly more often than males. This does not necessarily imply that females are more susceptible to ozone. This study found interesting but inconsistent trends regarding sex difference, including an indication that females may be at lower risk for IHD but higher risk for OHD for recent years (since 2008). By contrast, females were always at lower risk for CEV.

Some studies reported no sex-specific differences for ischemic stroke by case-crossover analysis [42], for cardiovascular disease by time series analysis [48], and for stroke in emergency department visits [49]. However, there is no clear evidence of sex-specific difference in ozone-hospitalization associations due to inconsistent reports. We agree with the view of the U.S. EPA in 2013: the lack of evidence for effect measure modification by sex may be indicative of the lack of association with cardiovascular morbidity, not the lack of an effect by sex [38]. Even if the sex-specific differences are statistically insignificant (within 0.2%), the study findings suggest further sex-specific studies are necessary to better understand the sex difference in reaction to ozone regarding circulatory hospitalizations.

### 4.4. Limitations of the Study

A limitation of this study is that individual level data are missing, as this was an ecologic study. For example, the same exposure to ozone was assumed for all residents within given CDs, and thus misclassification of ozone exposure is inevitable [50,51,52]. There also exist non-negligible discrepancies between ambient and personal ozone concentrations. For the warm season, personal-level ozone exposure would be modified by air conditioner usages [53,54], which were not controlled in the model.

## 5. Conclusions

In relation to ground-level ozone, this study examined hospital admissions with three specific types of circulatory causes—ischemic heart disease, other heart disease and cerebrovascular disease—which taken together comprise 85% of circulatory hospitalizations. While an overall decreasing trend was apparent for OHD, a mixed trend of incline (until the year 2008) and then decline for recent years appeared for IHD and CEV. This study reports not only a well-established finding that warm season 1-day lagged ozone is more highly associated with the three examined circulatory hospitalizations than cold season ozone, but also inconsistent sex specific differences in the association. In particular, this study found males to be at higher risks for IHD and females at higher risk for OHD; these findings have not been studied rigorously yet. The study findings could reduce the knowledge gap by identifying sub-populations susceptible to ozone by season, age, sex and trend in relation to circulatory hospitalizations. Further research on other causes such as respiratory related hospital admissions is warranted to fully understand the trends in ozone’s adverse health effects.

This study was supported through ‘Canadian Environmental Sustainability Indicators’ funding from Environment and Climate Change Canada (project 859104) and ‘Clean Air Regulatory Agenda’ funding from Health Canada (project 810523).

## Figures and Tables

**Table 1:** Demographic statistics for 24 Census Divisions.

City Name (East to West)	Pop. in 1000 1996–2012 (Trend %) ^1^	Pop. 50 ≤ Age < 65 in 1000 1996–2012 (Trend %) ^1^	Pop. Aged > 65 in 1000 1996–2012 (Trend %) ^1^	Female Aged > 65 in 1000 1996–2012 (Trend %) ^1^	Males Aged > 65 in 1000 1996–2012 (Trend %) ^1^
Halifax	351–407 (16)	47–85 (81)	36–54 (51)	14–24 (64)	21–30 (43)
Saint John	74–70 (−4)	10–15 (47)	11–12 (4)	4–5 (12)	7–7 (−1)
Quebec	513–567 (11)	83–103 (52)	65–103 (59)	24–42 (78)	40–60 (48)
Montreal	1798–1941 (8)	278–359 (29)	266–299 (12)	101–123 (21)	164–176 (7)
Ottawa	741–924 (25)	102–184 (80)	81–123 (52)	33–54 (64)	49–69 (43)
Durham	472–636 (35)	59–130 (120)	42–79 (87)	18–35 (94)	24–44 (81)
York	611–1086 (78)	85–219 (159)	50–131 (164)	22–60 (176)	28–71 (154)
Toronto	2456–2741 (12)	350–512 (46)	324–390 (20)	135–166 (23)	189–224 (18)
Peel	879–1365 (55)	116–255 (120)	63–146 (134)	27–67 (148)	36–80 (123)
Halton	350–528 (51)	54–99 (85)	38–71 (85)	17–31 (87)	22–40 (84)
Hamilton	481–541 (12)	69–111 (61)	67–85 (26)	28–37 (31)	39–48 (23)
Niagara	415–444 (7)	64–97 (52)	65–85 (29)	28–37 (35)	38–47 (26)
Waterloo	417–530 (27)	54–100 (86)	45–67 (49)	18–29 (62)	27–37 (41)
Windsor	204–218 (7)	28–43 (51)	29–35 (18)	12–15 (28)	18–20 (12)
Sarnia	75–74 (−1)	11–17 (51)	11–14 (29)	5–6 (33)	7–8 (26)
London	335–382 (14)	44–76 (73)	41–56 (37)	17–24 (44)	25–32 (32)
Sudbury	170–165 (−3)	25–36 (44)	20–27 (35)	9–12 (36)	11–15 (35)
Sault Ste. Marie	82–76 (−7)	13–18 (37)	11–15 (33)	5–7 (37)	6–9 (30)
Winnipeg	629–690 (10)	83–135 (62)	85–98 (15)	34–41 (23)	52–57 (9)
Regina	186–206 (11)	23–39 (62)	22–27 (22)	9–11 (27)	13–16 (18)
Saskatoon	199–239 (20)	23–43 (86)	23–29 (29)	9–12 (33)	14–17 (27)
Calgary	789–1179 (50)	95–217 (129)	69–116 (67)	29–51 (75)	40–65 (62)
Edmonton	632–869 (38)	80–159 (98)	69–99 (44)	29–43 (49)	40–56 (41)
Vancouver	1905–2408 (26)	263–483 (84)	221–327 (48)	93–149 (60)	128–178 (40)

^1^ Trend: (pop in 2012 − pop in 1996)/(pop in 1996) × 100.

**Table 2:** Statistics on circulatory hospitalization for 24 Census Divisions.

City Name	Number in 1000s of Circ 1996–2012 (trend, %) ^1^	Number in 1000s of IHD 1996–2012 (trend, %) ^1^	Number in 1000s of OHD 1996–2012 (trend, %) ^1^	Number in 1000s of CEV 1996–2012 (trend, %) ^1^
Halifax	4.1–2.9 (−28)	1.8–1.1 (−39)	1.05–1.02 (−3)	0.5–0.4 (−24)
Saint John	1.2–0.9 (−25)	0.4–0.3 (−36)	0.3–0.4 (23)	0.2–0.1 (−50)
Quebec	8.3–6.8 (−18)	3.2–2.2 (−31)	1.9–2.5 (34)	1.3–0.7 (−48)
Montreal	29.2–20.5 (−30)	10.0–6.0 (−40)	7.2–7.4 (2)	5.1–3.2 (−38)
Ottawa	7.8–6.0 (−23)	3.4–2.1 (−39)	1.8–2.3 (27)	1.3–0.9 (−30)
Durham	3.8–2.9 (−22)	1.5–0.9 (−38)	1.13–1.15 (2)	0.53–0.52 (−0.2)
York	3.0–4.5 (51)	1.1–1.7 (57)	0.9–1.7 (87)	0.6–0.7 (17)
Toronto	32.0–22.3 (−30)	12.7 –7.8 (−38)	8.9–8.4 (−5)	5.0–3.2 (−36)
Peel	5.0–8.0 (61)	1.8–3.3 (78)	1.3–2.6 (94)	0.8–1.1 (34)
Halton	3.5–2.9 (−17)	1.3–0.9 (−35)	1.1–1.3 (16)	0.5–0.4 (−20)
Hamilton	7.1–5.5 (−22)	3.1–1.7 (−47)	1.9–2.3 (25)	1.0–0.8 (−22)
Niagara	6.7–4.1 (−39)	2.6–1.4 (−46)	2.1–1.6 (−22)	1.0–0.6 (−38)
Waterloo	4.2–2.9 (−31)	1.4–1.1 (−25)	1.3–1.0 (−18)	0.7–0.5 (−30)
Windsor	3.3–2.0 (−40)	1.0–0.7( −31)	1.1–0.7 (−30)	0. 7–0.3 (−54)
Sarnia	1.4–0.9 (−38)	0.6–0.3 (−51)	0.42–0.36 (−13)	0.2–0.1 (−47)
London	3.6–2.9 (−20)	1.4–0.8 (−41)	0.9–1.1 (24)	0. 6–0.5 (−16)
Sudbury	2.8–2.0 (−29)	1.2–0.8 (−30)	0.67–0.68 (2)	0.3–0.2 (−32)
Sault Ste. Marie	1.2–1.1 (−9)	0.5–0.4 (−20)	0.4–0.5 (17)	0.2–0.1 (−17)
Winnipeg	7.8–5.7 (−26)	2.7–2.0 (−25)	2.2–2.0(−8)	1.5–0.9 (−35)
Regina	1.8–2.1 (17)	0.6–0.5 (−16)	0.7–1.0 (44)	0.2–0.3 (21)
Saskatoon	2.6–2.2 (−16)	0.9–0.6 (−27)	0.8–0.9 (11)	0.4–0. 3 (−31)
Calgary	7.4–7.2 (−2)	2.9–2.3 (−21)	2.1–2.8 (32)	0.99–1.03 (4)
Edmonton	5.4–5.3 (−1)	2.1–1.6 (−20)	1.3–1.9 (55)	0.8–0.9 (13)
Vancouver	21.5–18.0 (−16)	7.7–5.6 (−28)	6.71–6.75 (1)	3.4–3.0 (−11)

^1^ Trend: (counts in 2012 − counts in 1996)/(counts in 1996) × 100.

**Table 3:** Statistics for ozone and temperature.

City Name	Number of NAPS ^1^	Data Availability (%) ^2^	Ozone Range (ppb)		Temperature Range (°C)	
			Mean ^3^	Range ^4^	Mean ^5^	Range ^6^
Halifax	5	87.4	30.2	23.4–35.8	13.4	12.5–14.9
Saint John	4	100	33.0	29.2–37.4	12.5	11.5–13.5
Quebec	6	100	33.9	29.8–38.8	14.4	12.8–15.7
Montreal	14	100	35.0	29.8–39.4	16.4	15.0–17.4
Ottawa	3	99.4	36.2	27.2–41.2	16.1	14.9–17.3
Durham	2	96.8	41.4	36.7–47.6	15.6	14.3–16.9
York	2	93.2	47.4	42.5–53.3	15.9	14.4–17.0
Toronto	11	99.9	41.6	35.3–46.2	16.9	15.1–18.2
Peel	4	98.0	44.1	40.7–47.8	16.5	14.3–18.0
Halton	3	99.8	45.1	40.6–51.1	16.6	15.2–17.8
Hamilton	4	98.6	43.6	37.5–50.5	16.2	14.9–17.3
Niagara	2	95.7	45.2	38.5–51.7	16.9	15.4–18.0
Waterloo	2	97.3	46.1	41.4–53.2	15.6	14.1–16.8
Windsor	3	98.6	47.9	40.3–54.2	17.9	16.4–19.2
Sarnia	2	97.6	47.0	40.1–53.1	16.6	15.2–17.9
London	3	98.0	46.7	40.9–53.5	16.4	14.6–17.5
Sudbury	2	98.0	41.3	36.6–46.4	14.0	12.8–15.3
Sault Ste. Marie	3	99.6	40.8	37.1–43.7	12.8	11.1–14.0
Winnipeg	2	99.9	33.4	28.1–40.1	15.4	13.3–17.0
Regina	2	97.7	34.9	25.4–40.4	13.1	11.5–14.4
Saskatoon	1	92.8	31.5	20.7–39.5	13.2	11.8–14.6
Calgary	4	100	36.1	32.3–40.3	11.0	9.8–12.3
Edmonton	9	100	38.8	33.6–43.0	12.1	11.1–14.1
Vancouver	21	100	28.9	26.5–31.0	14.5	13.4–15.9

^1^ Number of NAPS monitoring stations included over 1996–2012. ^2^ Rate of non-imputed ozone availability: (number of daily ozone)/(total number of days). ^3^ Averaged daily 8-hour maximum ozone concentrations for warm months over 1996–2012. ^4^ Minimum and maximum of daily 8-hour maximum ozone concentrations for warm months over 1996–2012. ^5^ Averaged daily mean temperature for warm months over 1996–2012. ^6^ Minimum and maximum of daily mean temperature for warm months over 1996–2012.

## Supplementary Materials

The following are available online at http://www.mdpi.com/1660-4601/15/8/1566/s1, Figure S1: Over50 group. 95% posterior intervals for the associations per 10 ppb 1-day lagged ozone with IHD hospitalizations for warm season: (a) 17 years combined risks (square in orange); (b) 11 annual risks (circles in red), Figure S2: Senior group. 95% posterior intervals for the associations per 10 ppb 1-day lagged ozone with IHD hospitalizations for warm season: (a) 17 years combined risks (square in orange); (b) 11 annual risks (circles in red), Figure S3: Female group. 95% posterior intervals for the associations per 10 ppb 1-day lagged ozone with IHD hospitalizations for warm season: (a) 17 years combined risks (square in orange); (b) 11 annual risks (circles in red), Figure S4: Male group. 95% posterior intervals for the associations per 10 ppb 1-day lagged ozone with IHD hospitalizations for warm season: (a) 17 years combined risks (square in orange); (b) 11 annual risks (circles in red), Figure S5: Over50 group. 95% posterior intervals for the associations per 10 ppb 1-day lagged ozone with OHD hospitalizations for warm season: (a) 17 years combined risks (square in orange); (b) 11 annual risks (circles in red), Figure S6: Senior group. 95% posterior intervals for the associations per 10 ppb 1-day lagged ozone with OHD hospitalizations for warm season: (a) 17 years combined risks (square in orange); (b) 11 annual risks (circles in red), Figure S7: Female group. 95% posterior intervals for the associations per 10 ppb 1-day lagged ozone with OHD hospitalizations for warm season: (a) 17 years combined risks (square in orange); (b) 11 annual risks (circles in red), Figure S8: Male group. 95% posterior intervals for the associations per 10 ppb 1-day lagged ozone with OHD hospitalizations for warm season: (a) 17 years combined risks (square in orange); (b) 11 annual risks (circles in red), Figure S9: Base group. 95% posterior intervals for the associations per 10 ppb 1-day lagged ozone with CEV hospitalizations for warm season: (a) 17 years combined risks (square in orange); (b) 11 annual risks (circles in red), Figure S10: Over50 group. 95% posterior intervals for the associations per 10 ppb 1-day lagged ozone with CEV hospitalizations for warm season: (a) 17 years combined risks (square in orange); (b) 11 annual risks (circles in red), Figure S11: Senior group. 95% posterior intervals for the associations per 10 ppb 1-day lagged ozone with CEV hospitalizations for warm season: (a) 17 years combined risks (square in orange); (b) 11 annual risks (circles in red), Figure S12: Female group. 95% posterior intervals for the associations per 10 ppb 1-day lagged ozone with CEV hospitalizations for warm season: (a) 17 years combined risks (square in orange); (b) 11 annual risks (circles in red).
