# Supplementary material for: Air Health Trend Indicator: Association between Short-Term Exposure to Ground Ozone and Circulatory Hospitalizations in Canada for 17 Years, 1996–2012
Source: Int J Environ Res Public Health. 2018 Jul 24;15(8):1566. doi: 10.3390/ijerph15081566 (PMC6121235; doi:10.3390/ijerph15081566)
Supplement: Supplementary file 1 [file ijerph-15-01566-s001.pdf]

Supplementary Materials

In this appendix, we display a number of plots of the temporal evolution of 7-year weighted national posterior median associations between selected response variables (IHD hospitalizations and OHD hospitalizations) and a number of demographic sub-groups. In each case, the first displayed credible interval (yellow square) is for the entire time period (1996-2012 in all cases), while the following credible intervals (red circles) are for specific 7-year sub-groups.

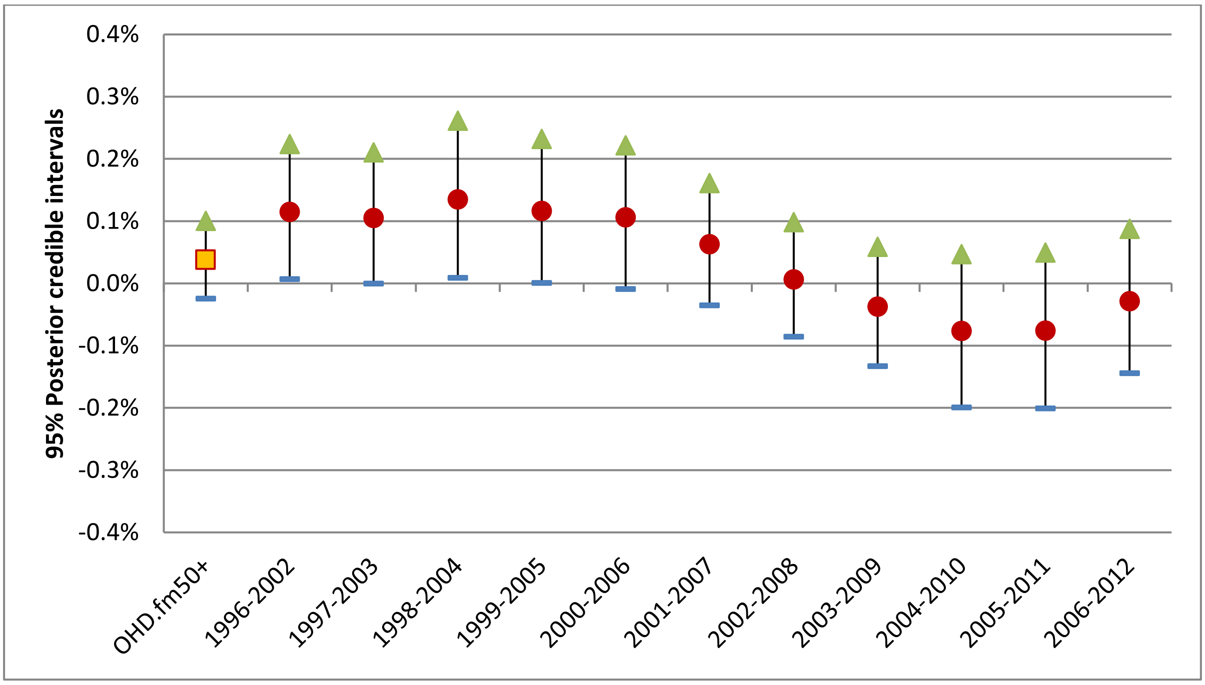

Figure S1: over50 group. 95% posterior intervals for the associations per 10 ppb 1-day lagged ozone with IHD hospitalizations for warm season: (a) 17 years combined risks (square in orange); (b) 11 annual risks (circles in red).

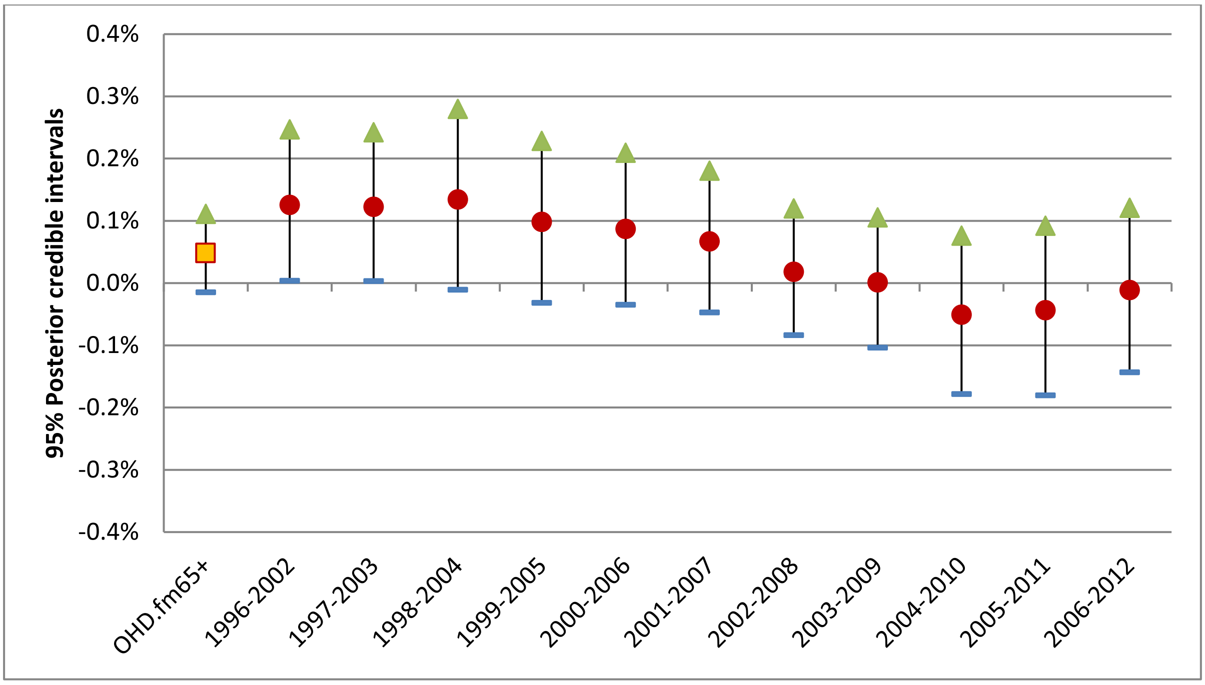

Figure S2: Senior group. 95% posterior intervals for the associations per 10 ppb 1-day lagged ozone with IHD hospitalizations for warm season: (a) 17 years combined risks (square in orange); (b) 11 annual risks (circles in red).

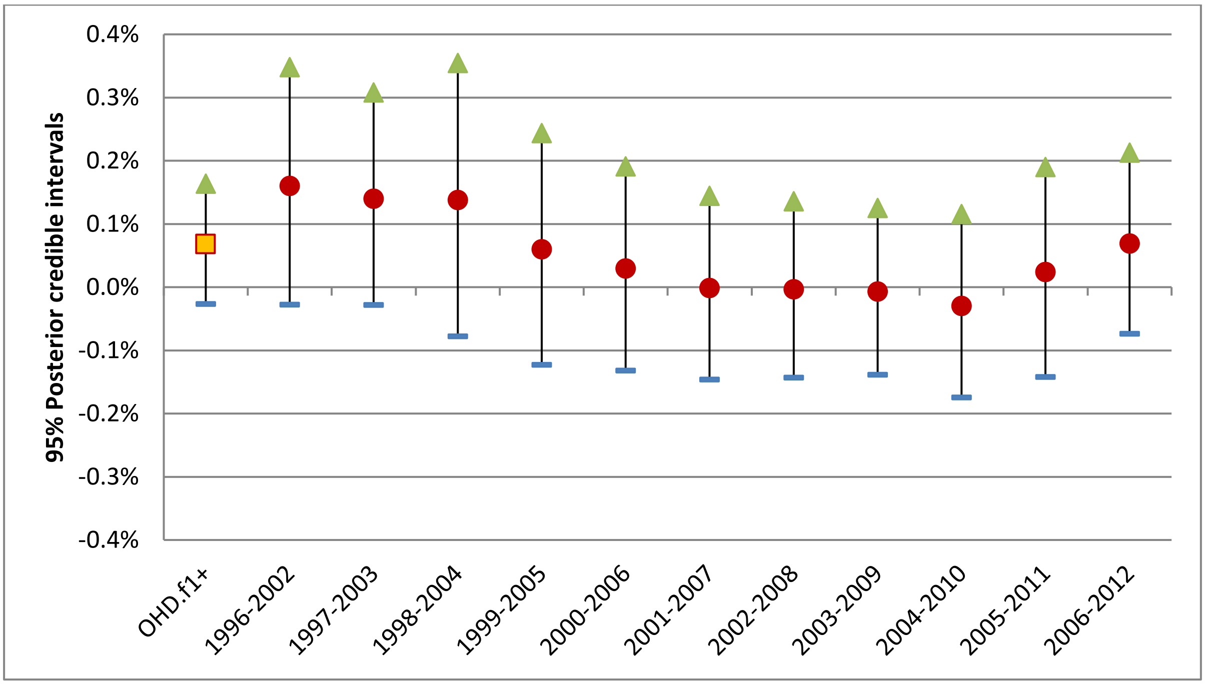

Figure S3: Female group. 95% posterior intervals for the associations per 10 ppb 1-day lagged ozone with IHD hospitalizations for warm season: (a) 17 years combined risks (square in orange); (b) 11 annual risks (circles in red).

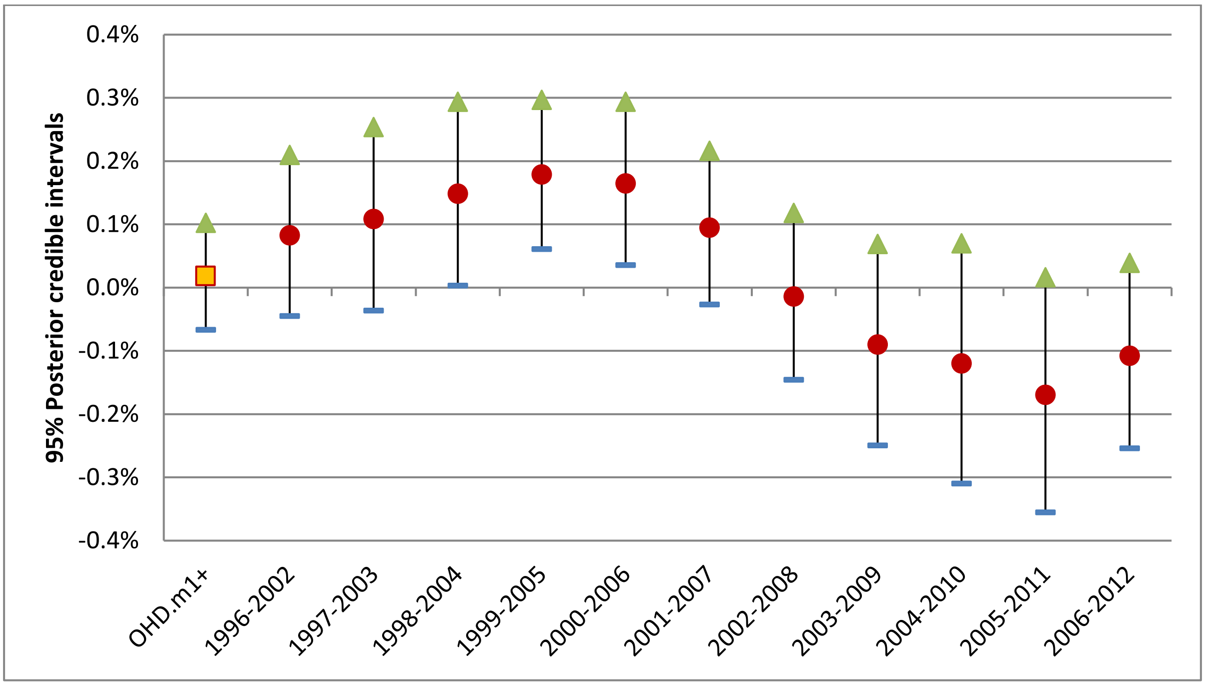

Figure S4: Male group. 95% posterior intervals for the associations per 10 ppb 1-day lagged ozone with IHD hospitalizations for warm season: (a) 17 years combined risks (square in orange); (b) 11 annual risks (circles in red).

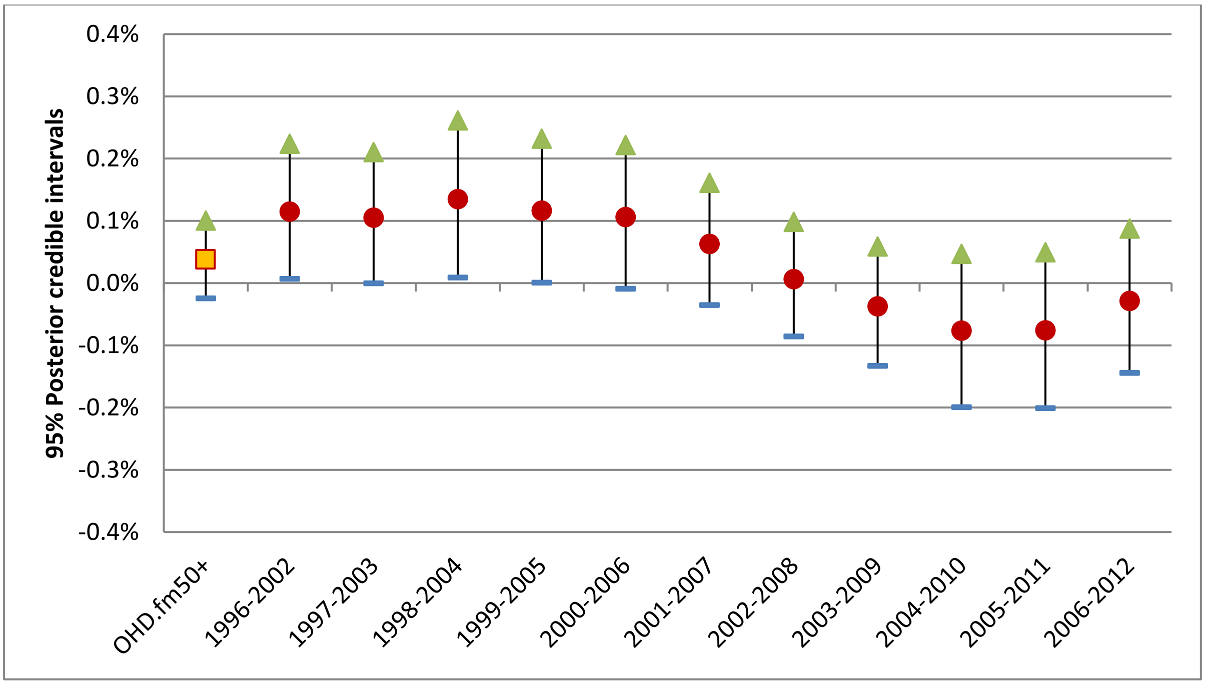

Figure S5: over50 group. 95% posterior intervals for the associations per 10 ppb 1-day lagged ozone with OHD hospitalizations for warm season: (a) 17 years combined risks (square in orange); (b) 11 annual risks (circles in red).

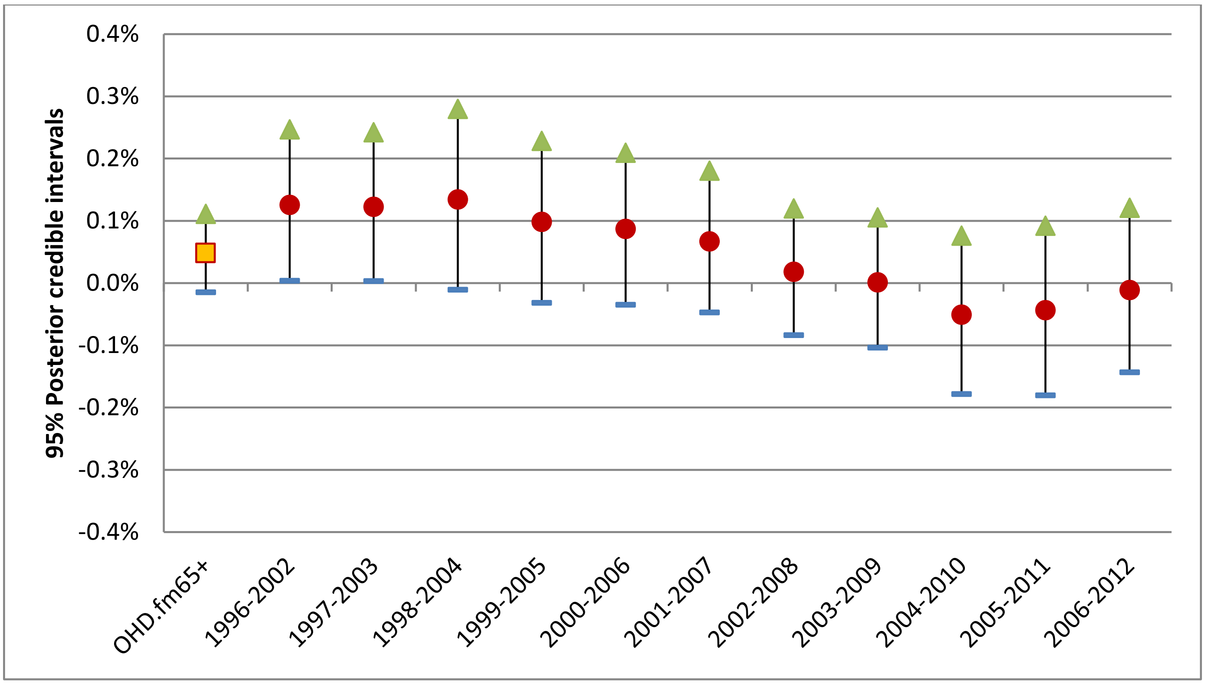

Figure S6: Senior group. 95% posterior intervals for the associations per 10 ppb 1-day lagged ozone with OHD hospitalizations for warm season: (a) 17 years combined risks (square in orange); (b) 11 annual risks (circles in red).

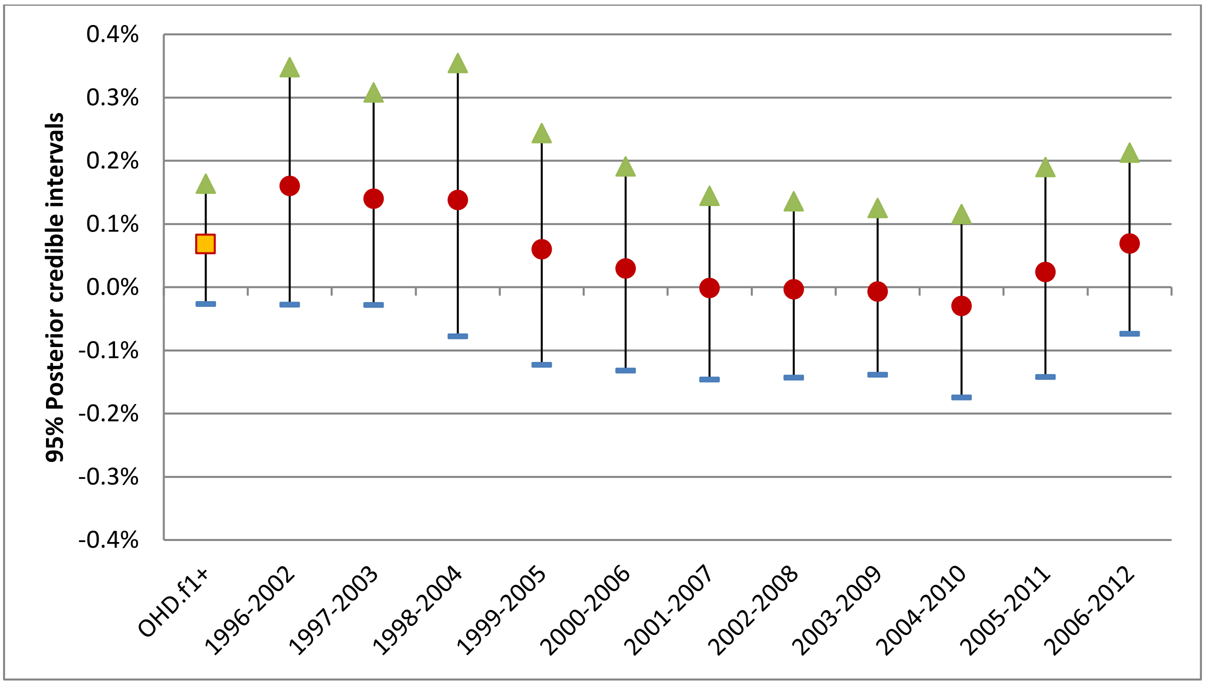

Figure S7: Female group. 95% posterior intervals for the associations per 10 ppb 1-day lagged ozone with OHD hospitalizations for warm season: (a) 17 years combined risks (square in orange); (b) 11 annual risks (circles in red).

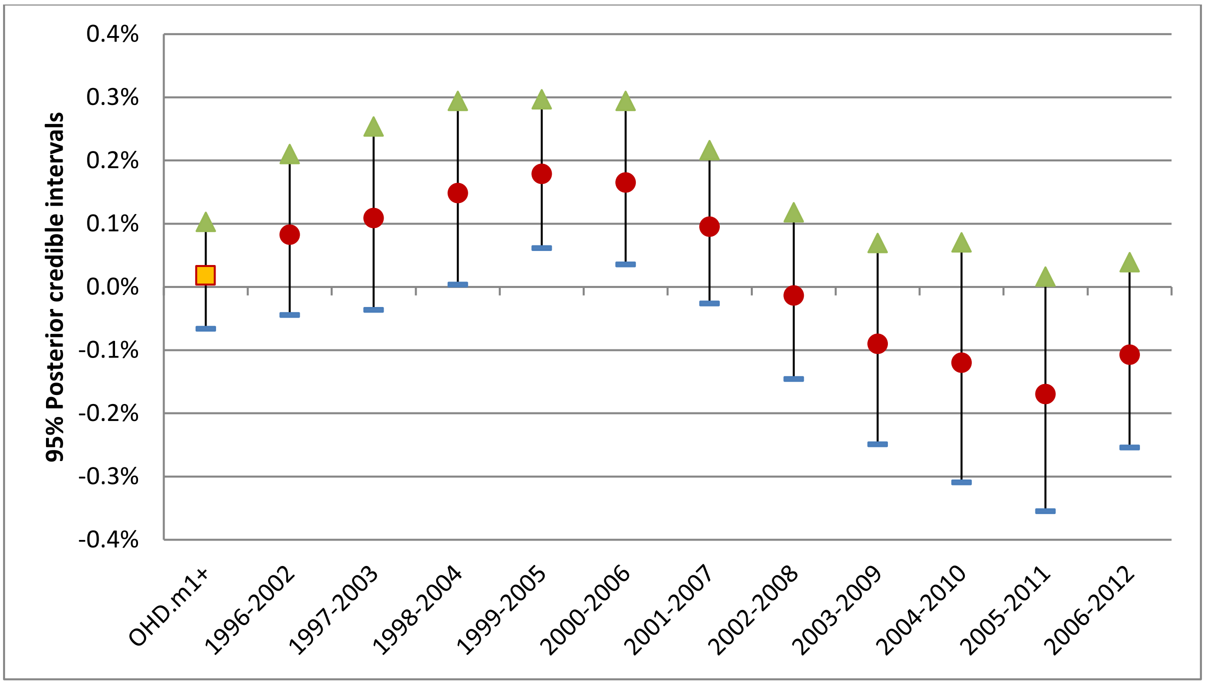

Figure S8: Male group. 95% posterior intervals for the associations per 10 ppb 1-day lagged ozone with OHD hospitalizations for warm season: (a) 17 years combined risks (square in orange); (b) 11 annual risks (circles in red).

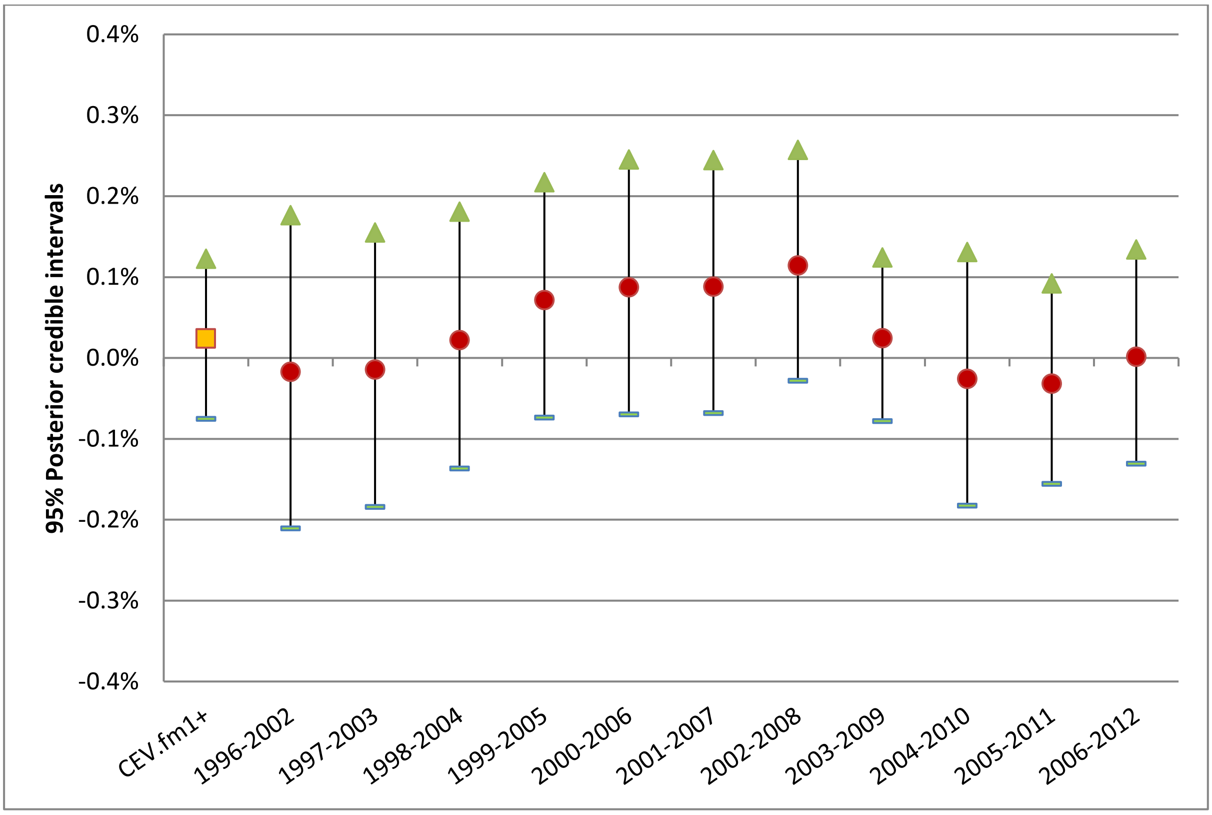

Figure S9: Base group. 95% posterior intervals for the associations per 10 ppb 1-day lagged ozone with CEV hospitalizations for warm season: (a) 17 years combined risks (square in orange); (b) 11 annual risks (circles in red).

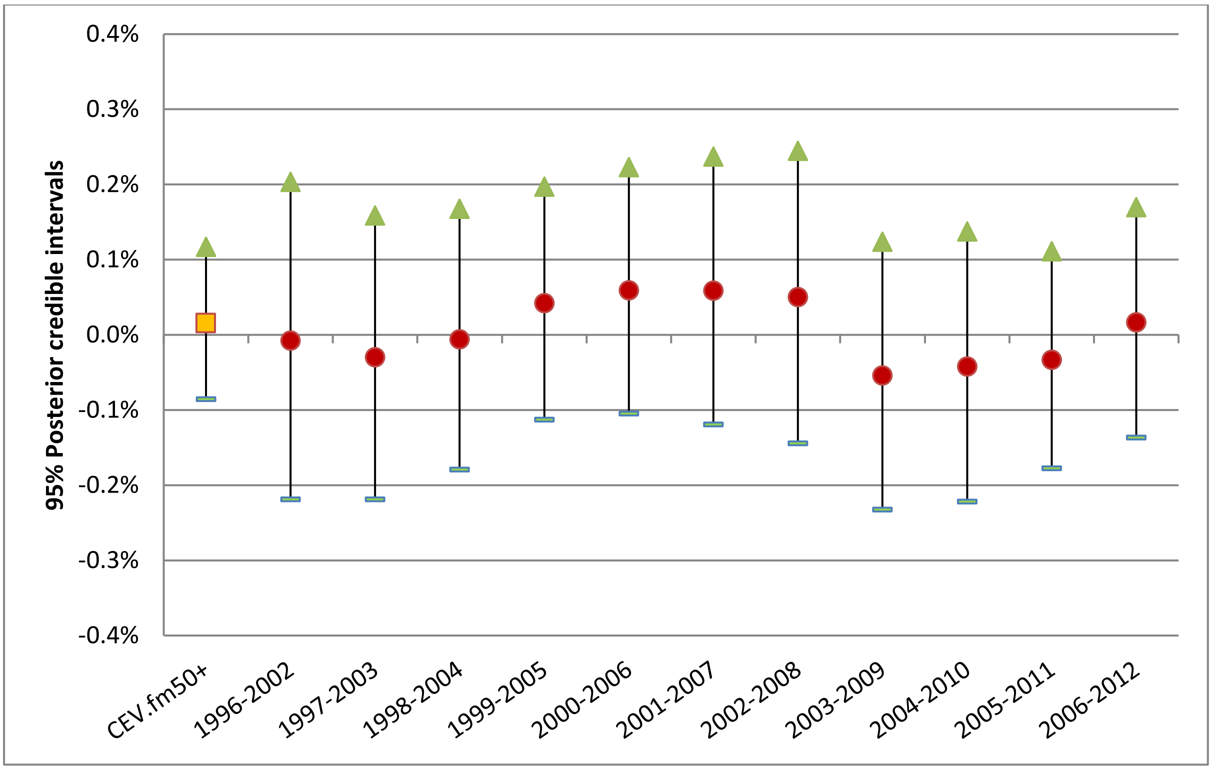

Figure S10: over50 group. 95% posterior intervals for the associations per 10 ppb 1-day lagged ozone with CEV hospitalizations for warm season: (a) 17 years combined risks (square in orange); (b) 11 annual risks (circles in red).

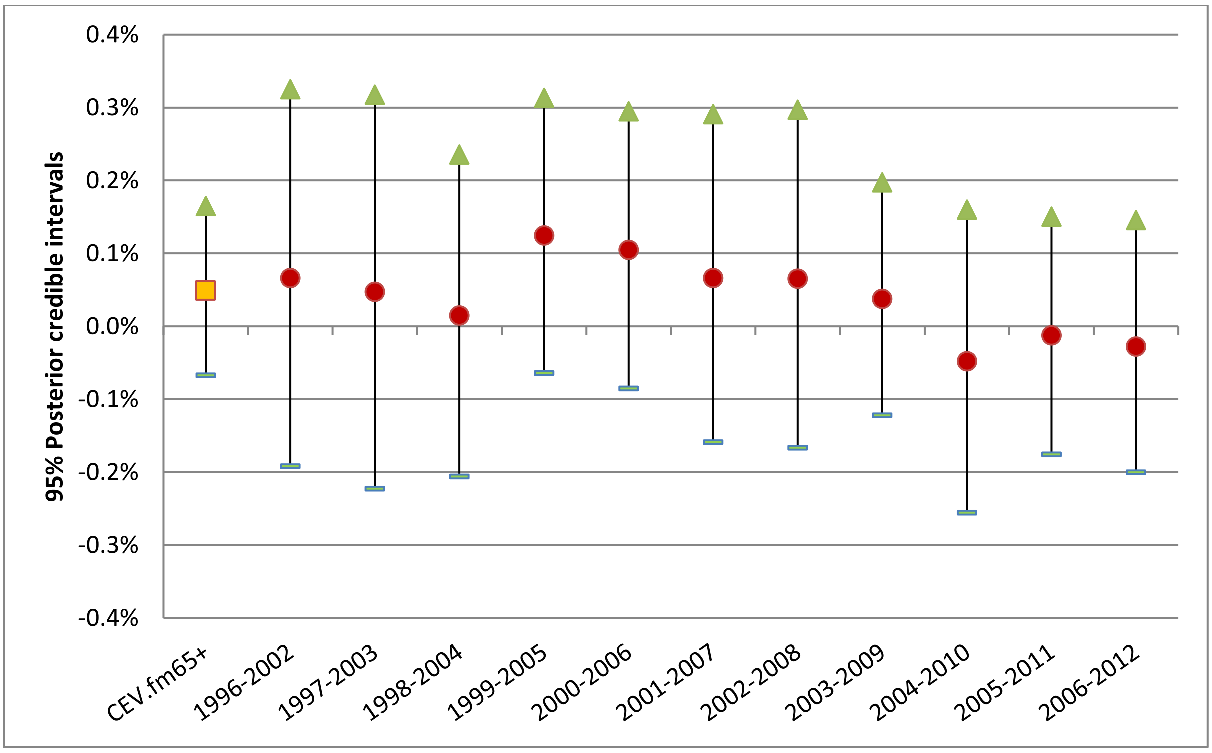

Figure S11: Senior group. 95% posterior intervals for the associations per 10 ppb 1-day lagged ozone with CEV hospitalizations for warm season: (a) 17 years combined risks (square in orange); (b) 11 annual risks (circles in red).

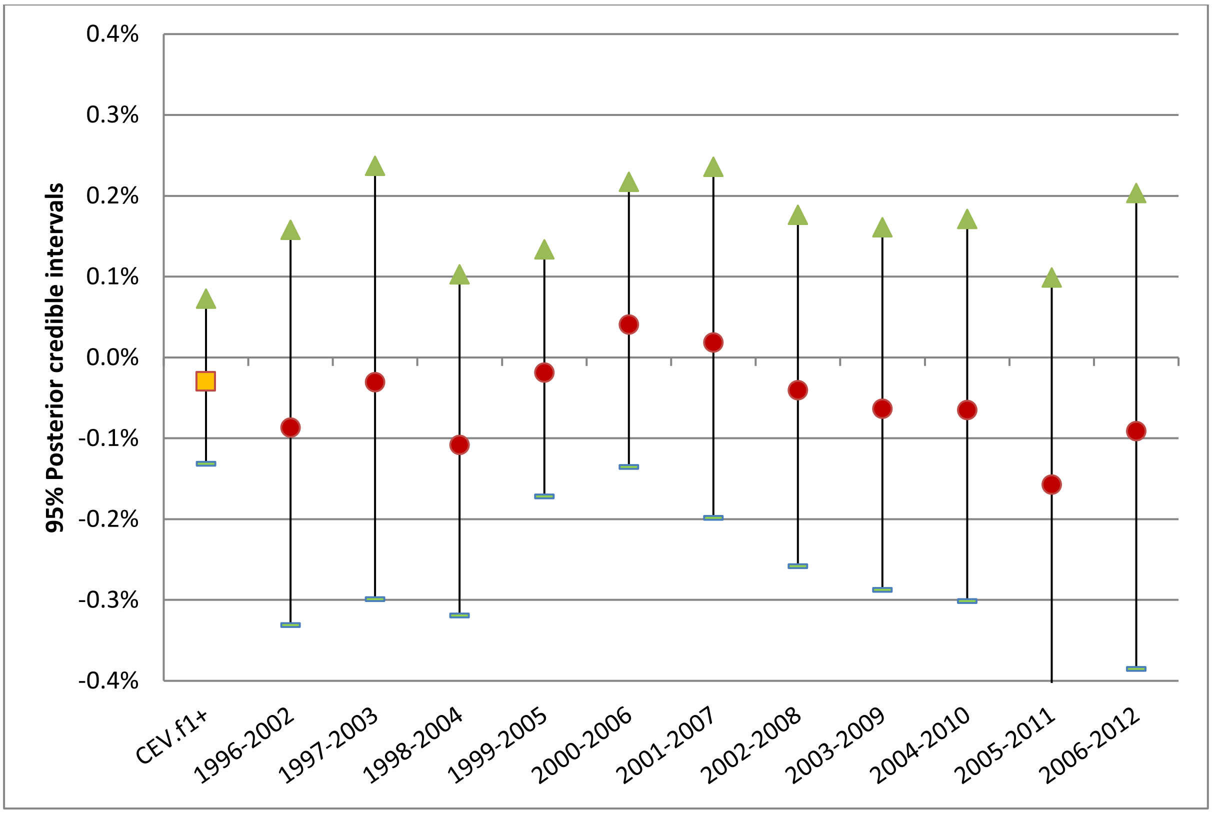

Figure S12: Female group. 95% posterior intervals for the associations per 10 ppb 1-day lagged ozone with CEV hospitalizations for warm season: (a) 17 years combined risks (square in orange); (b) 11 annual risks (circles in red).
